# Association of p53 Pro72Arg Polymorphism with Hepatocellular Carcinoma Risk in Hepatitis B Across Multiethnic Populations

**DOI:** 10.3390/cancers18030380

**Published:** 2026-01-26

**Authors:** Ulfa Kholili, Amal Arifi Hidayat, Ugroseno Yudho Bintoro, Soetjipto Soetjipto, Aryati Aryati, Alwi Alaydrus, Muhammad Miftahussurur

**Affiliations:** 1 Doctoral Program of Medical Science, Faculty of Medicine, Universitas Airlangga, Surabaya 60132, Indonesia; ulfa-k@fk.unair.ac.id; 2 Division of Gastroenterohepatology, Department of Internal Medicine, Faculty of Medicine, Universitas Airlangga, Surabaya 60132, Indonesia; amal.arifi.hidayat-2020@fk.unair.ac.id; 3 Helicobacter Pylori and Microbiota Study Group, Institute Tropical Disease, Universitas Airlangga, Surabaya 60115, Indonesia; 4 Division of Medical Oncology Hematology, Department of Internal Medicine, Faculty of Medicine, Universitas Airlangga, Surabaya 60132, Indonesia; 5 Department of Medical Biochemistry, Faculty of Medicine, Universitas Airlangga, Surabaya 60132, Indonesia; soetjiptoo@fk.unair.ac.id; 6 Department of Clinical Pathology, Faculty of Medicine, Universitas Airlangga, Surabaya 60132, Indonesia; aryati@fk.unair.ac.id; 7 Internal Medicine Specialist Study Program, Department of Internal Medicine, Faculty of Medicine, Universitas Airlangga, Surabaya 60132, Indonesia; muhammad.alwi.alaydrus-2021@fk.unair.ac.id; 8 Internal Medicine Specialist Study Program, Department of Internal Medicine, Dr. Soetomo General Academic Hospital, Surabaya 60286, Indonesia; 9 Department of Internal Medicine, Universitas Airlangga Hospital, Surabaya 60115, Indonesia

**Keywords:** hepatocellular carcinoma, hepatitis, p53 Pro72Arg polymorphism, multiethnic analysis, Non-Communicable Diseases

## Abstract

Introduction: Mounting evidence indicates that the p53 Pro72Arg single-nucleotide polymorphisms (SNPs) may play a role in modulating hepatocarcinogenesis in the setting of chronic HBV infection. However, there is currently a lack of studies focusing on this genetic variant in Indonesia, a country characterized by its diverse genetic landscape comprising over 1300 distinct ethnic groups. We aimed to investigate the association between the p53 Pro72Arg polymorphism and the risk of hepatocellular carcinoma (HCC) among Indonesian patients with chronic HBV infection. Methods: A total of 140 patients with chronic hepatitis B (CHB) were recruited, including 79 with HCC and 61 without HCC serving as controls. We used direct sequencing of DNA extracted from peripheral blood to analyze the SNPs of p53 codon 72. Results: The distribution of p53 Pro72Arg genotypes among Indonesian CHB patients was 12.9% for proline homozygotes (Pro/Pro), 31.4% for arginine homozygotes (Arg/Arg), and 55.7% for proline/arginine heterozygotes (Pro/Arg). Despite the lack of association between the SNPs and HCC risk in the overall population, both the Pro/Arg and Arg/Arg genotypes demonstrate an increased susceptibility to HCC compared to Pro/Pro genotypes exclusively in the Madurese ethnic group. Additionally, we discovered that in those with decompensated cirrhosis, the heterozygote Pro/Arg was more likely to develop HCC than the homozygous Pro/Pro. No significant association was found between the SNPs of p53 Pro72Arg and the clinicopathological characteristics of HCC. Conclusions: The p53 Pro72Arg polymorphism might contribute to hepatocarcinogenesis in Indonesian chronic hepatitis B patients, particularly Madurese and those with liver decompensation.

## Simple Summary

Hepatocellular carcinoma (HCC) is a complex, multistage disease influenced by viral and genetic factors. The tumor suppressor *p53 gene*, located on chromosome 17p13.1, plays a central role in genome protection through cell cycle arrest, apoptosis, senescence, and Deoxyribonucleic Acid (DNA) repair. A common single-nucleotide polymorphism at codon 72 (Pro72Arg, rs1042522) alters p53 function, with the arginine variant favoring apoptosis and the proline variant enhancing cell cycle arrest. To investigate its role in the diverse multiethnic population of Indonesia, we analyzed Pro72Arg polymorphism on 140 Chronic Hepatitis B (CHB) patients (79 with HCC, 61 controls) by direct DNA sequencing. Genotype frequencies were 12.9% Pro/Pro, 31.4% Arg/Arg, and 55.7% Pro/Arg. No overall association with HCC risk was observed; however, Madurese patients carrying Pro/Arg or Arg/Arg genotypes showed increased susceptibility, and Pro/Arg carriers with decompensated cirrhosis were more likely to develop HCC. These findings highlight ethnic and clinical subgroups where p53 Pro72Arg may contribute to hepatocarcinogenesis.

## 1. Introduction

Hepatocellular carcinoma (HCC), the most prevalent primary malignancy of the liver, constitutes a major global health concern. HCC is characterized by its rising incidence, frequent late-stage diagnosis, and limited therapeutic efficacy, culminating in a dismal prognosis [1]. Chronic infection with hepatitis B virus (HBV) stands as a paramount etiological factor in the pathogenesis of HCC [2], particularly in regions with high HBV endemicity. HBV initiates a cascade of events encompassing persistent liver inflammation, fibrosis, cirrhosis, and ultimately, malignant transformation of hepatocytes [3]. More than 50% of global HCC cases and 70–80% of HCC cases in regions endemic to hepatitis B are associated with HBV infection [4,5,6,7]. Indonesia has an intermediate to high endemicity of HBV infection, with prevalence ranging from 4.7% to 11.2% across different geographical areas. A multicenter study reported that over 60% of HCC cases in Indonesia are related to HBV infection [8].

The development of HCC is a complex, progressive, multiple-stage event driven by various factors. Not all individuals exposed to HBV infection develop HCC [9], indicating the significance of genetic factors in cancer risk. Several genes have been reported to be associated with the risk of HCC, including the tumor suppressor *p53 gene*. The *p53 gene*, located on chromosome 17p13.1, encodes a nuclear phosphoprotein that serves as a critical guardian of the genome [10]. As a master regulator, p53 induces cell cycle arrest, apoptosis, senescence, and DNA repair, while also inhibiting angiogenesis and metastasis [11]. The loss of p53 function has been identified as a key factor in hepatocarcinogenesis, putting p53 as an important candidate for predicting the risk of HCC [12]. Aside from mutations, various single-nucleotide polymorphisms (SNPs) have been identified in the *p53 gene* altering the biological function of the p53 protein. The SNP at codon 72, known as the Pro72Arg polymorphism (rs1042522), is one of the most extensively documented *p53 genetic* variants in different types of cancer, including HCC [13,14,15]. The p53 Arg72Pro polymorphism has also been implicated in increased susceptibility to several cancers such as lung, breast, colorectal, and cervical malignancies [16]. The p53 Pro72Arg polymorphism is a C/G variation upstream of the *p53 gene* resulting in three distinct genotypes: homozygous CC (Pro/Pro), homozygous GG (Arg/Arg), or heterozygous CG (Pro/Arg) [17]. Previous study indicated that the arginine variant excels at inducing apoptosis more rapidly and efficiently compared to the proline variant. In contrast, the Pro variant demonstrates a greater capacity for inducing cell cycle arrest [18].

The molecular pathogenesis of HCC related to hepatitis B significantly differs from HCC associated with other etiologies. One such mechanism is that the oncogene protein of hepatitis B virus X (HBx), which is essential for HBV replication, may disrupt the activity of the p53 protein by binding to it, sequestering it in the cytoplasm, and blocking its translocation to the nucleus, thereby preventing the activation of pro-apoptotic gene transcription [19]. Hence, it is important to explore HBV-related HCC separately from other etiologies to further understand the interplay between genetic variants of p53 and chronic HBV infection in the development of HCC. Indonesia, the fourth most populous country worldwide, features over 1300 distinct ethnic groups [20], resulting in significant potential for variation in genetic backgrounds. Despite this rich genetic landscape, there are currently no studies specifically investigating the *p53 gene* polymorphism in patients with HCC in Indonesia. Our study primarily aimed to investigate the association between the p53 Pro72Arg polymorphism and the risk of HCC development among patients with chronic hepatitis B in Indonesia. The secondary objective was to evaluate demographic and clinical factors as potential modifiers of the effect of p53 Pro72Arg genetic variants on HCC risk and to determine whether the clinicopathological characteristics of HCC were modified by the genotype profiles of p53 codon 72.

## 2. Methods

### 2.1. Study Design and Population

This is a single-center case–control study conducted at Dr. Soetomo General Academic Hospital, Indonesia, from September 2023 to January 2025. The minimum required sample size was estimated using the case–control formula with 95% confidence and 80% power, assuming an exposure prevalence of 50% among cases and 25% among controls. This yielded a requirement of at least 110 subjects (55 per group) [21]. The study included 140 chronic hepatitis B patients, comprising 79 with HCC and 61 without HCC as a control. HCC was diagnosed based on the guideline proposed by the Asian Pacific Association for the Study of the Liver (APASL) [22]. The exclusion criteria were HCV/HIV coinfection, pregnant women, and history of other malignancies, chemotherapy or systemic therapy, and autoimmune diseases.

Subjects were interviewed using a structured questionnaire to collect demographic and clinical information. The HBeAg status and AFP value were obtained from the most recent surveillance data recorded in electronic medical records. Multiphase CT was performed on all subjects, with positive findings for HCC characterized by arterial enhancement followed by venous washout in the delayed portal/venous phase. The definition of advanced fibrosis was LSM F3–F4 as measured by transient elastography (FibroScan^®^, Navi Mumbai, Indian). In patients with ascites, fibrosis staging was inferred from clinical and radiological evidence of cirrhosis, acknowledging the limitation of elastography in this setting. Decompensated cirrhosis was defined as the presence of at least a single event of jaundice, encephalopathy, bleeding, and/or ascites in patients having advanced fibrosis. Peripheral blood samples were collected from subjects and frozen at −80 °C until analysis.

### 2.2. DNA Extraction

The QIAamp^®^ Blood Mini Kit (Qiagen, Redwood City, CA, USA) was utilized for DNA extraction following the manufacturer’s protocols. Samples were added to protease and buffer AL, followed by incubation at 56 °C for a duration of 10 min. In brief, 200 μL of whole blood were incubated at 56 °C with proteinase K and 200 μL of Buffer AL for 15 min to lyse the cells and start protein digestion. Following incubation, 200 μL of 95% ethanol was added to promote DNA binding. The mixture was then transferred to a QIAamp spin column (QIAGEN GmbH, Hilden, Germany), centrifuged to bind the DNA to the membrane, and the supernatant discarded. The column was subsequently washed sequentially with Buffer AW1 and Buffer AW2 to remove residual impurities. Finally, the purified DNA was eluted using 200 μL of Buffer AE.

### 2.3. Genotyping of p53 Pro72Arg

The p53 Arg72Pro polymorphism (rs1042522), located within exon 4 of the *TP53 gene*, was selected owing to its established functional impacts on apoptotic regulation and cell cycle control, both of which are pivotal determinants of carcinogenesis. Bioinformatics analyses, including evolutionary conservation profiling and structural modeling, further substantiate its biological significance and reinforce its relevance as a candidate marker in genetic association studies [13,23]. Our study used specific primer pairs designed previously to target exon 2–4 of the *p53 gene* for polymerase chain reaction (PCR) amplification: forward primer (5′-CCCACTTTTCCTCTTGCAGC-3′), reverse primer (5′-CACTGACAGGAAGCCAAAGG-3′) [24]. The PCR reaction involved an initial denaturation step at 94 °C for 5 min, followed by 35 cycles of denaturation at 94 °C for 30 s, annealing at 61 °C for 30 s, and extension at 72 °C for 35 s. A final extension step was performed at 72 °C for 7 min. The PCR product was electrophoresed on a 1.5% ethidium bromide-stained agarose gel. After amplification, the DNA sequences were analyzed using an automated DNA sequencer (ABI 3730 DNA Analyzer; Applied Biosystems, Foster City, CA, USA).

### 2.4. Statistical Analysis

All data analyses were performed using IBM SPSS Statistics version 25.0 (IBM Co., Armonk, NY, USA). Median and interquartile range (IQR) were used to represent continuous data, while counts and percentages were used to represent categorical data. The distributions of subject characteristics between the HCC group and controls were assessed using the Mann–Whitney U test for continuous variables and the χ^2^ test for categorical variables. Genotype frequencies observed were compared to those expected, determined by applying the Hardy–Weinberg equilibrium equation (p^2^ + 2pq + q^2^ = 1; where p denotes the frequency of the wild-type allele and q denotes the frequency of the variant allele). A χ^2^ test was performed to evaluate deviations from Hardy–Weinberg equilibrium in both cases and controls. Pearson’s χ^2^ test was subsequently utilized to evaluate the significance of differences in genotype frequencies between HCC and control group. The relative risks were quantified as odds ratios (OR) by the calculation of ORs and their 95% confidence intervals (CIs) using binary logistic regression analysis, adjusted for age, sex, ethnicity, as well as the presence of advanced fibrosis and decompensated cirrhosis. Statistical modeling was implemented to evaluate the relative risk of the Arg/Arg or Pro/Arg genotypes in comparison to the Pro/Pro genotype as the reference. Additional statistical modeling was carried out to determine the dominant or recessive effect of the p53 Pro72Arg variants. The model comprising the relative risk of the Arg/Arg + Pro/Arg genotype against the Pro/Pro genotype (dominant model) or the Arg/Arg genotype against the Pro/Arg + Pro/Pro genotype (recessive model). We also performed a subgroup analysis to explore whether epidemiological factors and clinical parameters alter the risk of HCC across the p53 Pro72Arg polymorphism genotypes. In addition, the association between the genetic variants of p53 Pro72Arg and clinicopathological features of HBV-related HCC was analyzed using the χ^2^ test. Statistical significance was determined at a probability level of <0.05.

## 3. Results

### 3.1. Subject Characteristics

The general characteristics of subjects were summarized in Table 1. The study population primarily consisted of subjects aged 40 to 50 years, with a predominance of males. No statistically significant differences in age and sex were observed between HCC cases and controls. The Javanese represent the most prevalent ethnicity, followed by the Madurese and Chinese. Interestingly, specific ethnicities were found to have an association with the risk of HBV-related HCC. In Javanese patients, the rate of HCC among hepatitis B patients was lower compared to other ethnic groups (47.4% vs. 76.7%, *p* < 0.05). Contrarily, Madurese patients with chronic hepatitis B had a higher risk of developing HCC than the Javanese and Chinese patients combined (78.4% vs. 48.5%, *p* < 0.05). Smoking status, family history of HCC, and body mass index (BMI) did not indicate any significant differences. The complete absence of alcohol consumption in the control group deterred the comparative analysis. Over three-quarters of subjects in both groups were HBeAg negative. Our study population largely comprised patients with advanced fibrosis (LSM F3–F4); however the decompensation rate was comparable in both groups, at approximately 50%. No significant differences were observed in the status of HBeAg, advanced fibrosis, or decompensation between HCC cases and controls. Alpha-fetoprotein (AFP) levels were significantly higher in patients with HCC compared to those without (2000.0 vs. 36.0, *p* < 0.05), as expected.

### 3.2. Variants of the p53 gene Polymorphism in Exons 2–4

**Figure 1:**
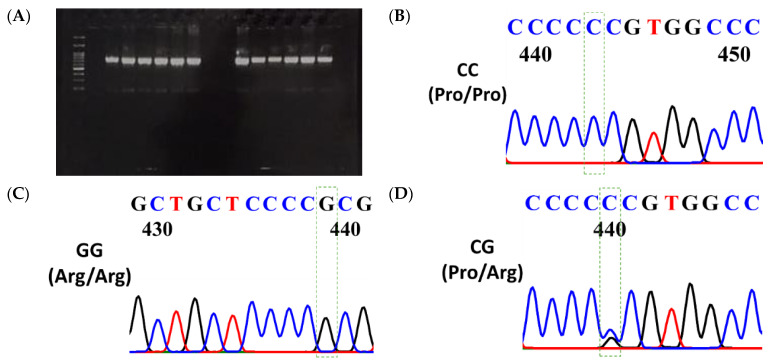
(**A**) Electropherogram of the PCR products using primer pairs covering Exons 2–4 (764 base pairs). The sequencing result presented as (**B**) CC (Pro/Pro, single peak, homozygote), (**C**) GG (Arg/Arg, single peak, homozygote), and (**D**) CG (Pro/Arg, double peaks, heterozygote), respectively. The uncropped blots are shown in Figure S1. Electropherogram color coding: Blue = Cytosine (C), Red = Thymine (T), Black = Guanine (G). Green wireframe = The position of p53 Pro72Arg polymorphism in the electropherogram.

The p53 genomic DNA reference sequence was retrieved from the NCBI database (association number: NC_000017.10). Figure 1A displays the electropherogram of PCR products (764 bps). Our results identified four single-nucleotide polymorphisms (SNPs) and nucleotide losses in exons 2–4 of the *p53 gene*. The present study is focused on codon 72 polymorphism (rs1042522), positioned at the 119th base of exon 4. Based on the reference sequence, it was expected that this spot would contain a C nucleotide (CC, homozygote, single peak; see Figure 1B). However, in various samples, it was either a G (GG, homozygote, single peak; see Figure 1C) or a CG (heterozygote, two peaks; see Figure 1D). The other SNPs were found in the third intron (between exons 3–4), exon 4, and the second intron (between exons 2–3), respectively. The losses of the 16-bp duplication polymorphism were found in the third intron of the *p53 gene*. The detailed results for these will be reported elsewhere.

### 3.3. p53 Arg72Pro Polymorphism and HCC Risk

Table 2 depicts the genotype frequency distribution of p53 Pro72Arg polymorphism. The heterozygous genotype CG (Pro/Arg) is the most prevalent across the overall population (55.7%) and the HCC group (60.8%), followed by the homozygous genotypes GG (Arg/Arg) and CC (Pro/Pro). Both the HCC and control groups had genotype frequencies consistent with Hardy–Weinberg equilibrium (χ^2^ = 5.343, df = 2, *p* = 0.068; χ^2^ = 0.018, df = 2, *p* = 0.991), indicating the absence of population stratification and sampling bias. Moreover, the distribution of genotype frequencies in the HCC group was comparable to that of the controls (χ^2^ = 0.4238, df = 2, *p* = 0.121).

Despite the risk not being statistically significant, the heterozygotes (Pro/Arg genotype) were more likely to develop HCC (OR_adj_ = 1.985; 95% CI = 0.655–6.013; *p* = 0.226). The risk of HCC was similar between subjects with two G alleles and those with CC genotypes (OR_adj_ = 1.081; 95% CI = 0.334–3.503; *p* = 0.226). In reference to the Pro/Pro genotypes, the combined genotypes of Pro/Arg and Arg/Arg in the dominant model demonstrated a non-significant increase in the risk of HCC (OR_adj_ = 1.584; 95% CI = 0.547–4.587; *p* = 0.397). We additionally observed a non-significantly higher risk of HCC in homozygote Arg/Arg compared to combinations of Pro/Pro and Pro/Arg variants in the recessive model (OR_adj_ = 1.878; 95% CI = 0.904–3.901; *p* = 0.091).

### 3.4. Subgroup Analysis

A subgroup analysis was conducted to explore potential modification of the effect of p53 Pro72Arg polymorphisms by demographic aspects, including sex and ethnicity (Table 3). No statistically significant association was found between p53 Pro72Arg polymorphism and the risk of HCC in male patients. The null value in homozygotes CC precluded the comparison among female patients. Subanalysis by ethnicity revealed no significant differences in HCC risk across the three genotypes in Javanese patients. Interestingly, among the Madurese ethnic group, the heterozygote CG (Pro/Arg) demonstrated higher risk of HCC compared to CC (Pro/Pro) genotype (OR_adj_ = 38.722; 95% CI = 1.628–920.939; *p* = 0.024). Furthermore, Madurese patients carrying two G alleles had a 25-fold increased likelihood of HCC relative to homozygotes CC (OR_adj_ = 25.269; 95% CI = 1.179–541.732; *p* = 0.039). Subanalysis within the Chinese ethnic group was not feasible due to inadequate sample size.

We also performed a stratified analysis based on clinical parameters associated with the development of HCC in chronic hepatitis B, such as HBeAg status, advancing fibrosis, and decompensation (Table 4). In both HBeAg positive and HBeAg negative populations, there was no significant association between p53 Pro72Arg genotypes and the HCC risk. In individuals with advanced fibrosis, the CG and GG genotypes demonstrated a non-significant elevated risk of HCC compared to those with two C alleles. A similar non-significant association was observed in chronic hepatitis B patients with no advanced fibrosis. Intriguingly, in individuals with decompensated cirrhosis, the CG heterozygote was associated with a significantly increased risk of HCC. These subjects were eight times more likely to develop HCC compared to those with the CC genotype (OR_adj_ = 8.027; 95% CI = 1.149–56.087; *p* = 0.036). In contrast, no statistically significant associations were observed across the three genotypes in the subpopulation with no liver decompensation.

### 3.5. p53 Arg72Pro Polymorphism and Clinicopathological Features of HBV-Related HCC

The association between p53 Pro72Arg genetic variants and clinicopathological features of the cancer was further investigated in the HCC cohort (See Table 5). No statistically significant differences were observed across genotypes regarding tumor number (single vs. multiple) and diameter (<5 vs. ≥5 cm). The p53 Pro72Arg polymorphism was additionally not related to the progression of HCC stage according to the Barcelona Clinic Liver (BCLC) system. Lastly, portal thrombosis and distant metastases were not significantly associated with any of the three genotypes.

## 4. Discussion

This investigation reported the very first genetic profile of p53 codon 72 polymorphisms in chronic hepatitis B patients, both with and without HCC, in Indonesia, a nation marked by significant ethnic diversity. Our study also provided evidence on the impact of p53 codon 72 polymorphisms on the risk of HCC development, specifically in the setting of chronic HBV infection. The importance of our findings lies in providing baseline genetic data for an understudied population, thereby contributing to the global understanding of host genetic factors in HBV-related hepatocarcinogenesis. Furthermore, we highlighted the clinical significance of ethnic origin and decompensation status in relation to susceptibility of HCC associated with the p53 Pro72Arg polymorphism among patients with chronic HBV infection in Indonesia. In chronic hepatitis B populations, as well as in those with HCC specifically, the most frequently observed genotypes were the heterozygotes Pro/Arg, followed by the homozygotes Arg/Arg and Pro/Pro. The genotype distribution in Indonesian population resembles that of Chinese and Turkish HCC cohorts, characterized by a predominance of heterozygotes [25,26]. Three independent study group involving HCC cohorts of Caucasian and African descent demonstrated a distinct distribution of p53 Pro72Arg genotypes, in which the homozygous Arg/Arg genotype emerging as the most prevalent [27,28,29]. The similar genotype distribution with other Asian cohorts could be explained by the fact that our study population comprised individuals with Austronesian ancestral lineage, specifically Javanese, Madurese, and Chinese. This study demonstrated no significant association between p53 Pro72Arg genotypes and the occurrence of HCC in Indonesian patients with chronic HBV infection. A prior meta-analysis involving 1511 HCC cases and 2165 controls indicated that the risk of HCC is lower in arginine-containing genotypes (Arg/Arg and Pro/Arg combined) compared to proline homozygotes (Pro/Pro) [30]. However, their stratified analysis, which only included patients with chronic HBV infection, failed to find any association between p53 Pro72Arg polymorphisms and HCC risk. A Turkish study reported conflicting results, suggesting that proline-rich genotypes (Pro/Pro) present a four-fold increased risk of HCC compared to the Arg/Arg genotype in patients with hepatitis B [26]. The conflicting results could stem from gene-environment interactions, where specific genotypes might interact with local risk factors to promote HCC development. Furthermore, differences in genetic ancestry and HBV strains could modulate the effect of the p53 polymorphism on HCC risk. Our results indicated that patients of Madurese origin had an increased likelihood of HCC, regardless of their p53 codon 72 variant status. Our findings align with previous studies that have demonstrated racial differences in HCC incidence [31,32]. Moreover, the risk of HCC in Asians was reported to be nearly double that of white Hispanics and more than four times higher than that of Caucasians [31]. Ethnicity can independently affect the risk of HCC due to genetic variations that predispose certain populations to liver disease and shape their immune response to HBV infections [33,34]. Additionally, ethnic groups often have distinct lifestyles, dietary habits, and exposure to environmental carcinogens, all of which contribute to varying HCC incidence rates.

Interestingly, despite no significant difference being observed in the overall Indonesian chronic hepatitis B population, a subanalysis focusing exclusively on the Madurese revealed that the p53 Pro72Arg polymorphism was associated with an increased susceptibility to HCC. In this ethnic group, we discovered that the Arg/Arg and Pro/Arg genotypes showed a higher risk of HCC development in comparison to the proline-rich genotype. The relevance of ethnicity as a potential modifier of the effect of p53 Pro72Arg polymorphism on HCC risk has been documented in previous meta-analysis [30]. It was found that a proline-rich genotype is associated with a higher risk of HCC in Asian populations than both Arg/Arg and Pro/Arg genotypes, while no significant difference was observed in the Caucasian race. In contrast to previous findings, our study indicates that the arginine-containing genotypes are associated with an increased likelihood of HCC in Madurese ethnic, rather than the proline-homozygous genotypes. The Pro/Pro genotype has often been associated with a higher risk of HCC due to lower p53 stability or altered interactions with proteins involved in DNA repair, apoptosis, or cell cycle control, particularly in the setting of HBV infection [35]. On the other hand, the increased apoptotic activity of the Arg72 variant, while generally tumor-suppressive, can paradoxically contribute to hepatocarcinogenesis in specific circumstances [34]. Chronic HBV infection and ongoing liver damage could result in higher apoptosis, which potentially exacerbates inflammation and tissue injury [36]. This feature may promote a microenvironment favorable for HCC development, induce compensatory cell proliferation, and increase the likelihood of genetic errors and malignant transformation. It is challenging to pinpoint a specific molecular mechanism for why the p53 72Arg variant might be associated with a higher HCC risk specifically in Madurese patients. The Madurese ethnic may have unique genetic variations that, in combination with the 72Arg variant, affect susceptibility to HCC. Furthermore, we postulated that dietary exposure to carcinogen could alter the potential link between the p53 Pro72Arg polymorphism and HCC risk, among the Madurese population. A dietary survey conducted in Madura reported that 11% of candlenuts sourced from a local traditional market were contaminated with *Aspergillus flavus*, the primary producer of the potent hepatocarcinogen Aflatoxin B1 [37]. Moreover, a separate investigation discovered that almost all levels of Aflatoxin B1 food contamination originating from Indonesia exceed the maximum regulatory limit [38]. The Arg72 variant may enhance aflatoxin-induced DNA damage through its pro-apoptotic activity, resulting in higher inflammation and an increased risk of HCC.

In our subgroup analysis stratified by HBeAg status, p53 Pro72Arg genotypes did not demonstrate a significant association with HCC risk. This observation differs from several reports in the literature, where findings have been inconsistent ranging from increased susceptibility to no detectable effect [14,39]. Such variability likely reflects differences in study design, sample size, ethnic background, and viral–host interactions. In subanalysis of patients with liver decompensation, we observed that the heterozygotes Pro/Arg had a significantly higher risk of HCC than the homozygous Pro/Pro, while the arginine-rich homozygotes did not. Decompensated cirrhosis is marked by substantial liver dysfunction, resulting in elevated cellular stress, inflammation, and DNA damage. In this setting, p53 activation is increased as it attempts to address the significant injury [40]. There may be no discernible effect of the Arg/Pro variations on HCC risk when p53 function is largely unaltered, as in compensated cirrhosis or in those without cirrhosis. In decompensated cirrhosis, however, the Pro/Arg heterozygote might represent a “tipping point”. The presence of both alleles could lead to a situation where the balance between apoptosis, DNA repair, and cell cycle control is disrupted in a way that promotes HCC specifically under conditions of increased stress. This study also revealed no significant associations between p53 Pro72Arg genotypes and any clinicopathological characteristics of HBV-related HCC. An earlier investigation in China reported findings consistent with ours regarding BCLC stage, satellite nodules, and vascular invasion [41]. Nevertheless, they observed that the combination of p53 codon 72 Pro/Pro and MDM2 G/G was associated with larger tumor diameter and the presence of unencapsulated tumors relative to other genotypes. Tumor size and encapsulation are complex traits influenced by multiple genetic and environmental factors, while the p53 polymorphism might have a subtle effect that is only detectable in specific contexts.

Given the nature of genetic association studies, there are several limitations to consider. Our limited sample size might restrict statistical power, potentially overlooking modest but significant associations. The observational design presents a challenge in establishing causality between the p53 Pro72Arg polymorphism and the risk of HCC. Also, there was a lack of representation from individuals of Melanesian descent in our study, who are the primary inhabitants of eastern Indonesia, particularly Papua Island. In fact, an earlier study of p53 codon 72 polymorphism profiles revealed that indigenous Papuan populations harbor a low frequency of the G (Arg) allele [42].

## 5. Conclusions

This study provided novel evidence that the p53 Pro72Arg polymorphism might contribute to hepatocarcinogenesis in Indonesian chronic hepatitis B populations, specifically individuals of Madurese ethnicity and those with liver decompensation. Future studies should focus on multi-ethnic analyses with larger sample sizes to validate existing findings and explore the nuanced impact of the p53 Pro72Arg gene polymorphism across diverse populations. Mechanistic studies are needed to elucidate the functional differences between the Arg and Pro variants and how they interact with HBV proteins and other cellular pathways involved in hepatocarcinogenesis. Additionally, incorporating environmental factors, such as aflatoxin exposure, and other genetic polymorphisms will provide a more comprehensive understanding of HCC risk. Ultimately, our study allowed a more tailored risk assessment strategy and targeted therapies for patients at increased risk of developing HCC due to chronic HBV infection.

## Figures and Tables

**Table 1:** Characteristics of study population.

	HCC (*n* = 79)	Controls (*n* = 61)	*p*
Age (years)	54.0 (13.0)	48.5 (12.5)	0.398
Male	66 (83.5%)	47 (77.0%)	0.334
Ethnicity			0.005 *
Javanese	46 (58.2%)	51 (83.6%)	
Madurese	29 (36.7%)	8 (13.1%)	
Chinese	4 (5.1%)	2 (3.3%)	
BMI (kg/m^2^)	21.5 (3.7)	21.0 (3.7)	0.918
NIDDM	9 (11.4%)	9 (14.8%)	0.556
Alcohol consumption	2 (2.5%)	0 (0%)	-
Smoking	9 (11.4%)	4 (6.6%)	0.328
Family history ^1^	3 (3.8%)	5 (8.2%)	0.266
HBeAg positive	19 (24.1%)	14 (23.0%)	0.879
Advanced fibrosis	63 (79.7%)	50 (82.0%)	0.741
Decompensated cirrhosis	42 (53.2%)	25 (41.0%)	0.153
AFP (ng/mL)	2000.0 (18,321.0)	36.0 (631.0)	0.002 *

* *p* < 0.05; ^1^ Family history of HCC in first-degree relatives. Abbreviations: AFP, alpha-fetoprotein; BMI, body mass index; NIDDM, non-insulin-dependent type 2 diabetes mellitus.

**Table 2:** Distribution of genotype frequency of p53 Pro72Arg polymorphism and its association with HCC risk.

	HCC (*n* = 79)	Controls (*n* = 61)	*p* ^1^	OR_adj_ (95% CI)
General genotype				
CC (Pro/Pro)	9 (11.4%)	9 (14.8%)		1.00 (Reference)
CG (Pro/Arg)	48 (60.8%)	30 (49.2%)	0.226	1.984 (0.655–6.013)
GG (Arg/Arg)	22 (27.8%)	22 (36.1%)	0.897	1.081 (0.334–3.503)
Dominant model				
CC (Pro/Pro)	9 (11.4%)	9 (14.8%)		1.00 (Reference)
CG (Pro/Arg) + GG (Arg/Arg)	70 (88.6%)	52 (85.2%)	0.397	1.584 (0.547–4.587)
Recessive model				
CC (Pro/Pro) + CG (Pro/Arg)	31 (39.2%)	31 (50.8%)		1.00 (Reference)
GG (Arg/Arg)	48 (60.8%)	30 (49.2%)	0.091	1.878 (0.904–3.901)

^1^ *p*-values were computed using logistic regression analysis.

**Table 3:** Analysis of the frequency distribution of p53 Pro72Arg genetic variants stratified by sex and ethnicity.

Subgroup	Genotype	*N*_case_	*N*_control_	*p* ^1^	OR_adj_ (95% CI)
Male		66	47		
	CC (Pro/Pro)	9 (13.6%)	8 (17.0%)		1.00 (Reference)
	CG (Pro/Arg)	38 (57.6%)	21 (44.7%)	0.267	1.948 (0.600–6.325)
	GG (Arg/Arg)	19 (28.8%)	18 (38.3%)	0.987	0.989 (0.284–3.451)
Female		13	14		
	CC (Pro/Pro)	0 (0.0%)	1 (7.1%)		-
	CG (Pro/Arg)	10 (76.9%)	9 (64.3%)	-	-
	GG (Arg/Arg)	3 (23.1%)	4 (28.6%)	-	-
Javanese		46	51		
	CC (Pro/Pro)	7 (15.2%)	5 (9.8%)		1.00 (Reference)
	CG (Pro/Arg)	29 (63.0%)	27 (52.9%)	0.841	0.871 (0.225–3.370)
	GG (Arg/Arg)	10 (21.8%)	19 (37.3%)	0.290	0.453 (0.104–1.966)
Madurese		29	8		
	CC (Pro/Pro)	2 (6.9%)	4 (50.0%)		1.00 (Reference)
	CG (Pro/Arg)	16 (55.2%)	2 (25.0%)	0.024 *	38.722 (1.628–920.939)
	GG (Arg/Arg)	11 (37.9%)	2 (25.0%)	0.039 *	25.269 (1.179–541.732)
Chinese		4	2		
	CC (Pro/Pro)	0 (0.0%)	0 (0.0%)		-
	CG (Pro/Arg)	1 (25.0%)	1 (50.0%)	-	-
	GG (Arg/Arg)	3 (75.0%)	1 (50.0%)	-	-

* p < 0.05; ^1^ *p*-value were computed using logistic regression analysis.

**Table 4:** Subgroup analysis of p53 Pro72Arg polymorphism genotypes based on HBeAg status, advanced fibrosis, and decompensated cirrhosis.

Subgroup	Genotype	*N*_case_	*N*_control_	*p* ^1^	OR_adj_ (95% CI)
HBeAg positive		19	14		
	CC (Pro/Pro)	4 (21.0%)	2 (14.3%)		1.00 (Reference)
	CG (Pro/Arg)	12 (63.2%)	5 (35.7%)	0.223	5.546 (0.352–87.447)
	GG (Arg/Arg)	3 (15.8%)	7 (50.0%)	0.735	0.613 (0.036–10.470)
HBeAg negative		60	47		
	CC (Pro/Pro)	5 (8.3%)	7 (14.9%)		1.00 (Reference)
	CG (Pro/Arg)	36 (60.0%)	25 (53.2%)	0.218	2.424 (0.593–9.909)
	GG (Arg/Arg)	19 (31.7%)	15 (31.9%)	0.370	2.001 (0.439–9.111)
Advanced fibrosis		63	50		
	CC (Pro/Pro)	6 (9.5%)	6 (12.0%)		1.00 (Reference)
	CG (Pro/Arg)	40 (63.5%)	26 (52.0%)	0.223	2.379 (0.590–9.598)
	GG (Arg/Arg)	17 (27.0%)	18 (36.0%)	0.608	1.471 (0.337–6.433)
No advanced fibrosis		16	11		
	CC (Pro/Pro)	3 (18.8%)	3 (27.3%)		1.00 (Reference)
	CG (Pro/Arg)	8 (50.0%)	4 (36.4%)	0.549	1.950 (0.220–17.285)
	GG (Arg/Arg)	5 (31.3%)	4 (36.4%)	0.390	4.257 (0.156–115.850)
Decompensation		42	25		
	CC (Pro/Pro)	4 (9.5%)	5 (20.0%)		1.00 (Reference)
	CG (Pro/Arg)	28 (66.7%)	13 (52.0%)	0.036 *	8.027 (1.149–56.087)
	GG (Arg/Arg)	10 (23.8%)	7 (28.0%)	0.297	2.812 (0.403–19.634)
No decompensation		37	36		
	CC (Pro/Pro)	5 (13.5%)	4 (11.1%)		1.00 (Reference)
	CG (Pro/Arg)	20 (54.1%)	17 (47.2%)	0.903	1.105 (0.224–5.437)
	GG (Arg/Arg)	12 (32.4%)	15 (41.7%)	0.631	0.668 (0.129–3.456)

* *p* < 0.05; ^1^ *p*-value were computed using logistic regression analysis.

**Table 5:** The genotype profiles of the p53 Pro72Arg polymorphism in relation to the clinicopathological features of HCC.

*n* = 79	CC (Pro/Pro)	CG (Pro/Arg)	GG (Arg/Arg)	*p* ^1^
Single tumor	6 (14.6%)	25 (61.0%)	10 (24.4%)	0.562
Multiple tumor	3 (7.9%)	23 (60.5%)	12 (31.6%)	
Tumor with diameter of <5 cm	1 (10.0%)	7 (70.0%)	2 (20.0%)	0.805
Tumor with diameter of ≥5 cm	8 (11.6%)	41 (59.4%)	20 (29.0%)	
BCLC B	6 (20.7%)	15 (51.7%)	8 (27.6%)	0.129
BCLC C-D	3 (6.0%)	33 (66.0%)	14 (28.0%)	
No portal thrombus	8 (15.7%)	28 (54.9%)	15 (29.4%)	0.195
Portal thrombus	1 (3.6%)	20 (71.4%)	7 (25.0%)	
No distant metastases	8 (13.6%)	36 (61.0%)	15 (25.4%)	0.483
Distant metastases	1 (5.0%)	12 (60.0%)	7 (35.0%)	

^1^ *p*-values were calculated from χ^2^ test. Abbreviation: BCLC, Barcelona Clinic Liver Cancer staging.

## Data Availability

The datasets generated and/or analyzed during this study are available in the Figshare repository and can be accessed via the following DOI: 10.6084/m9.figshare.30617531.

## Supplementary Materials

The following supporting information can be downloaded at: https://www.mdpi.com/article/10.3390/cancers18030380/s1, Figure S1: Western blots information.
