# Supplementary figures and images for: Association of p53 Pro72Arg Polymorphism with Hepatocellular Carcinoma Risk in Hepatitis B Across Multiethnic Populations
Source: Cancers (Basel). 2026 Jan 26;18(3):380. doi: 10.3390/cancers18030380 (PMC12896443; doi:10.3390/cancers18030380)

SUPPLEMENTARY FIGURE 1

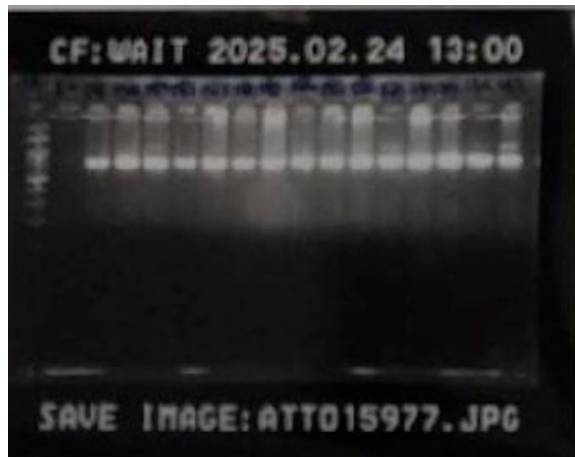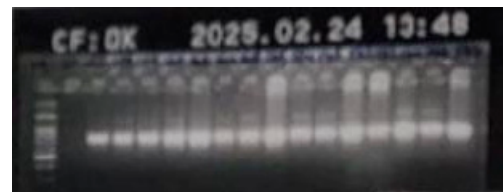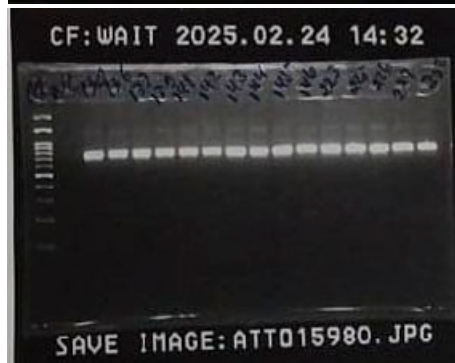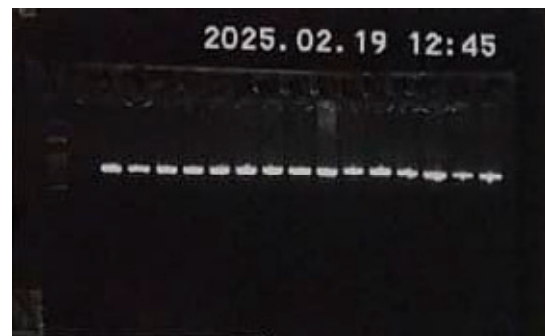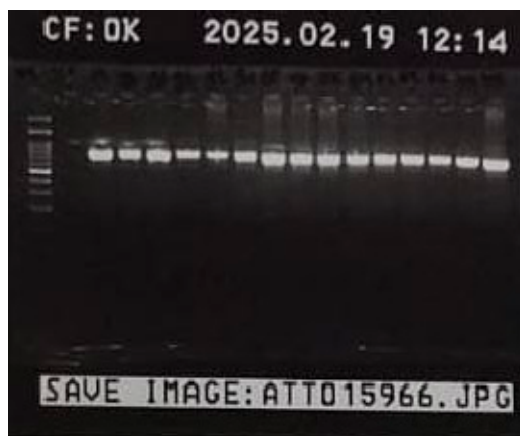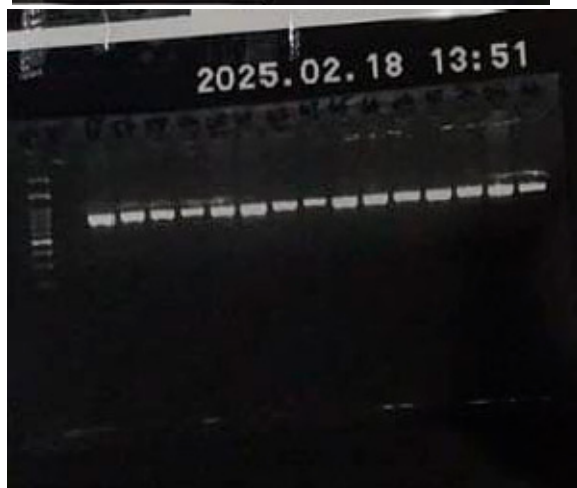

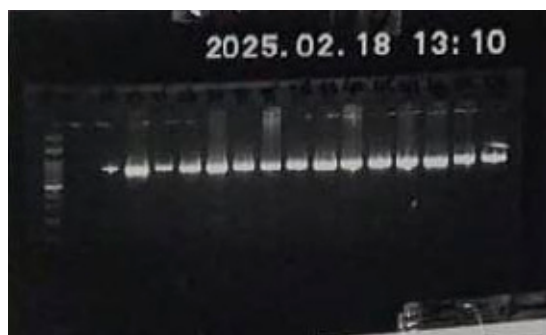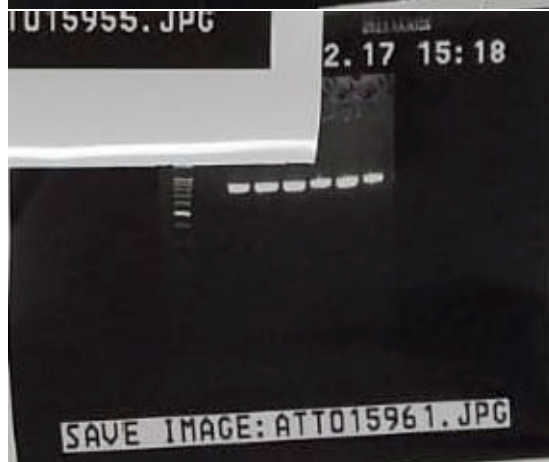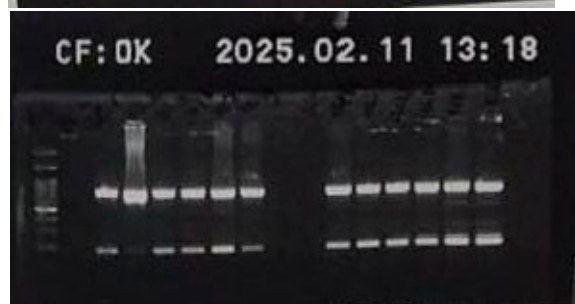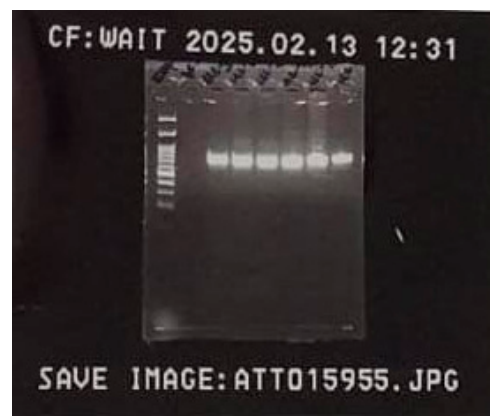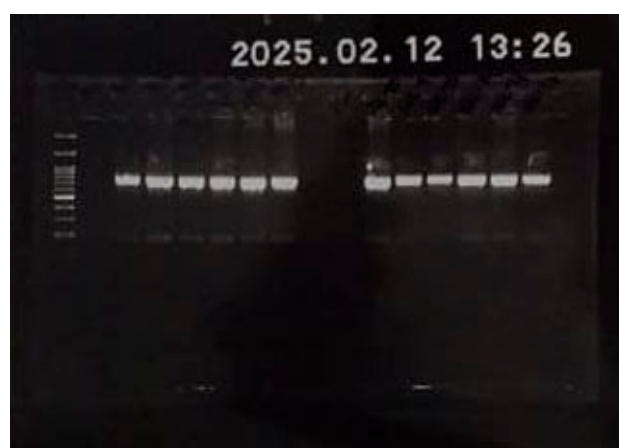

Supplement: Supplementary file 1 [file cancers-18-00380-s001.zip › cancers-4100655-supplementary.pdf]
